# LGBTQ+ cardiovascular health equity: a brief review

**DOI:** 10.3389/fcvm.2024.1350603

**Published:** 2024-03-06

**Authors:** Jason A. Bonomo, Kate Luo, Jorge A. Ramallo

**Affiliations:** ^1^ Inova Scar Heart and Vascular, Inova Health System, Falls Church, VA, United States; ^2^ University of Virginia School of Medicine, Charlottesville, VA, United States; ^3^ Inova Pride Clinic, Inova Health System, Falls Church, VA, United States

**Keywords:** LGBTQ+ cardiovascular health, health equity, social drivers of health, SOGI, cardiovascular risk

## Abstract

Cardiovascular disease (CVD) is the leading cause of morbidity and mortality in the United States. Data shows that social drivers of health (SDOH), including economic stability, racial/cultural identity, and community, have a significant impact on cardiovascular morbidity and mortality. LGBTQ+ (lesbian, gay, bisexual, transgender, queer, and other gender and sexual minority) patients face a variety of unique health risk factors and bear a disproportionate burden of CVD compared to cis-gender, heterosexual peers. There is a paucity of research assessing the etiologies of CVD health disparities within the LGBTQ+ community. Herein, we seek to explore existing literature on LGBTQ+ health disparities with a focus on cardiovascular disease, examine trends impacting LGBTQ+ health equity, and identify strategies and interventions that aim to promote LGBTQ+ cardiovascular health equity on a regional and national level.

## 1 Introduction to LGBTQ+ health

The lesbian, gay, bisexual, transgender, queer, and other gender and sexual minority population represents roughly 7.1% of the population in the United States (1). This heterogeneous community includes individuals who identify as lesbian, gay, bisexual, transgender, and queer, along with others who may identify with a different sexual orientation or gender identity (SOGI). On a whole, SOGI minority patients report lower overall self-reported rates of health compared to cis-gender heterosexual controls (2); a patient's SOGI intersect with other aspects of their identity including race, ethnicity, and other social drivers of health (SDOH) which can impact health outcomes. Over the past several decades, SDOH have been increasingly recognized as drivers of health inequity nationally (3).

Health equity refers to the concept of attaining the highest levels of health and wellness for all peoples; to understand one's set of unique set of health needs, data must exist which explore the intersection of SDOH and epidemiologic health data. Until recently, such data for LGBTQ+ patients were lacking. In 2011, the National Academy of Medicine released a report identifying these research gaps and called for research focused at better understanding LGBTQ+ specific health issues (4). More recently, the American Heart Association (AHA) released a statement in 2020 outlining ways to better understand LGBTQ+ cardiovascular health disparities, identify research gaps, while also providing a framework for research aimed at better understanding LGBTQ+ CV health equity (5).

## 2 LGBTQ+ health disparities

LGBTQ+ patients face health disparities on a regional and national level compared to their cis-gender peers in the United States. Many of these disparities are driven by inequities related to the community's SDOH. One of the most unifying long-term risk factors for adverse health outcomes in LGBTQ+ patients is related to minority stress (6). Minority stress is broadly driven by pervasive and chronic forms of stress borne by individuals who live in a society which marginalizes and discriminates against them (2, 6). Contributors to minority stress are multifactorial and include increased rates of discrimination at the familial, social, cultural, and employer level (7–9), amongst other sets of internal pressures, which result in higher rates of mental health disorders in the LGBTQ+ population and, putatively, higher rates of physiologic stress (2, 6, 10, 11). SOGI minority patients living in states which practice institutionalized discrimination against LGBTQ+ peoples face higher rates of mental illness compared to those living in states without such policies (12). Increased levels of psychosocial stressors result in increased physiological stress, an adverse impact on the immune system, and hypertension (13–15). The physiological impact of minority stress on the LGBTQ+ population have not been well studied (16), although it has been extensively studied in non-LGBTQ+ minority populations.

While minority stress is a common unifier of health inequity within the LGBTQ+ community, subgroups within the LGBTQ+ community face unique risk factors that impact overall health and well-being. Gay and bisexual men have higher rates of human immunodeficiency virus (HIV) (17). HIV contributes to chronic inflammation, and some drugs used in the treatment of HIV adversely affect cardiometabolic risk (18). Certain non-nucleoside reverse transcriptase inhibitors (NNRTIs) including stavudine and zidovudine have been shown to promote lipoatrophy (19). Protease inhibitors, including ritonavir, and other NNRTIs such as efavirenz and nevirapine, have been shown to promote dyslipidemia (19). Tenofovir lowers levels of high-density lipoproteins, low-density lipoproteins, and total cholesterol, but whether this translates into lower cardiovascular risk in setting of a chronic inflammatory milieu driven by HIV, is unknown (19). Highly active antiretroviral therapy also increases the risk for developing type 2 diabetes mellitus roughly four-fold (20).

Bisexual men face higher rates of substance abuse compared to both gay and straight men, and rates of substance abuse are generally higher in SOGI adults compared to the general population (21, 22). Lesbian women have higher rates of obesity and alcohol use compared to their heterosexual peers (23, 24). Moreover, lesbian women are more likely to engage in tobacco use compared to heterosexual women (25). Lesbian and bisexual women also have high rates of depression, anxiety, asthma and arthritis compared to heterosexual women (26, 27). Transgender patients are more likely to experience physical violence and have higher rates of housing instability and homelessness compared to heterosexual peers (28).

SOGI minority people of color (POC), particularly those with African ancestry, face increased rates of discrimination compared to white LGBTQ+ patients; this includes higher rates of social discrimination, racism, employment discrimination, and housing discrimination (29). This has led to higher rates of depression in LGBTQ+ POC compared to white LGBTQ+ peoples (30). Structural racism was associated with higher rates of anxiety and alcohol abuse for SOGI minority men of color, but not for white SOGI minority men (31). Gay and bisexual men who are black also face significantly higher rates of HIV and decreased utilization of pre-exposure prophylaxis compared to white gay and bisexual men (32). Roughly half (47%) of all SOGI patients of color live in a low-income household compared to 36% for white SOGI patients (33).

## 3 LGBTQ+ cardiovascular health disparities

LGBTQ+ patients also face increased risk for cardiovascular disease compared to the general population (34). Risk factors for cardiovascular disease generally exhibit wide variation across LGBTQ+ subgroups, and risk factors shared by one SOGI group may not be observed in other SOGI groups. However, on a whole, cardiovascular disease tends to develop at an earlier age for LGBTQ+ patients compared to heterosexual peers (35).

Observational studies have consistently shown that LGBTQ+ patients have higher rates of hypertension compared to their heterosexual peers (15, 35). Sexual minority women are diagnosed with hypertension roughly a decade earlier than heterosexual women, or between the ages of 35–44 (compared to 45–54 for heterosexual women) (35). Gay and bisexual men experience increased rates of hypertension compared to heterosexual men, but age at onset to diagnosis does not differ when compared to heterosexual men (35, 36). Transgender men also experience increases in blood pressure following gender affirming hormone therapy (GAHT), while for transgender women, blood pressure decreases with GAHT (37).

No concrete data exists as to the extent and mechanisms driving this observed risk of hypertension for SOGI minority patients. One hypothesis is that physiologic mediators of chronic stress (minority stress theory) may drive this disparity, which has been observed and studied in other cis-gender minority communities adversely impacted by chronic stress (38).

Gay and bisexual men, as well as lesbian and bisexual women, have higher rates of dyslipidemia compared to their heterosexual peers (35). Gender affirming hormone therapy (GAHT) has been shown to promote dyslipidemia in transgender patients, but evidence is mixed as to whether this translates into worse long-term cardiovascular outcomes (39, 40). HAART therapy in HIV + patients can promote dyslipidemia, and there are numerous drug-drug interactions between HAART and statin medications; the recent REPRIEVE trial showed that for patients with HIV and moderate or greater risk of cardiovascular disease, pitavastatin reduced rates of major adverse cardiovascular outcomes (41).

Lesbian, gay, and bisexual patients have higher rates of stroke compared to their heterosexual peers (35). Gay and bisexual men have roughly twice the adjusted risk of myocardial infarction and heart failure compared to heterosexual men (35). Transgender men face higher rates of myocardial infarction compared to both to cis-gender men and women (42). Clear mechanisms explaining this increased have not been identified. Despite having higher rates of heart disease, LGBTQ+ patients are less likely to be treated for primary prevention of heart disease with statin medications compared to their heterosexual peers, even after controlling for a variety of social and economic risk factors (43). The authors speculated that the residual disparities may be the result of “bias, stereotyping, and mistrust” (43).

The effects of GAHT on cardiac remodeling remains unknown, and robust data does not exist on the topic (44). Older data had suggested that transgender women had higher rates of venous thromboembolism (VTE) compared to the general population, but this observation was only with an estradiol no longer used with GAHT (39). More recent studies have not observed an increased risk of VTE in transgender men or women on GAHT under the age of 37 years old (45). Prospective cohort studies are needed to better understand what, if any, long term effects on cardiac remodeling or VTE are associated with GAHT.

**Figure 1:**
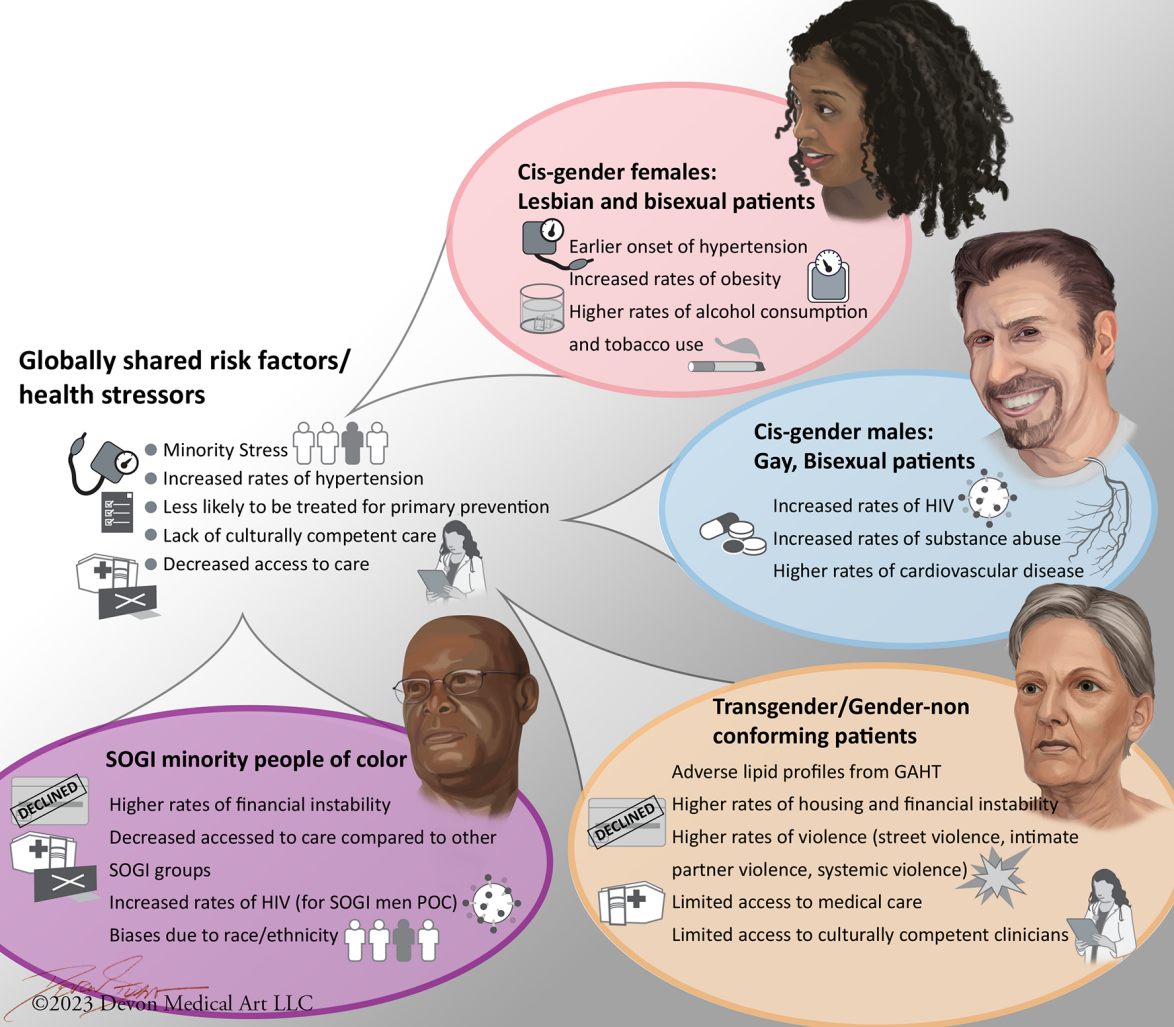
Heterogeneity of social drivers of health and health disparities within the LGBTQ+ population. Cis-gender refers to a patient whose gender identity corresponds with the sex assigned at birth. SOGI refers to sexual orientation and gender identity. While LGBTQ+ patients share some similar risk factors which increase their risk of cardiovascular disease, these disparities are not consistently observed between different LGBTQ+ subgroups.

Our current understanding of the unique health risks that affect LGBTQ+ patients is summarized in Figure 1. While there is some commonality to health disparities affecting LGBTQ+, there is significant heterogeneity within each community subgroup. Even within subgroups, there is significant variation in terms of health inequities, some of these being driven by one's SDOH (Figure 1).

## 4 LGBTQ+ barriers to care

Having access to quality, culturally competent health care is paramount component of SDOH. Numerous barriers to care for LGBTQ+ patients have been identified. These include structural discrimination, financial barriers to care, and lack of access to culturally competent care for LGBTQ+ patients in the United States.

SOGI minority patients have increasingly faced sociopolitical systemic discrimination in the United States, where a growing social milieu in areas of the United States has evolved to limit LGBTQ+ access to care (46, 47). This sociopolitical discrimination has been rising across the United States in recent years (48). For example, in Texas and Mississippi, LGBTQ+ focused University-based health centers were forced to close under political duress (49, 50). Prescribing GAHT, a treatment which reduces rates of depression and suicide attempts in transgender adolescents, is currently a felony in 5 US states (48). In 3 other states, it is illegal to provide GAHT although it is not considered a felony (48). 19 states within the US restrict GAHT care for adolescents, and 16 of the 19 state laws restricting access to GAHT have been passed within the past year (48). The United States House of Representatives recently passed a bill in July of 2023 which would eliminate fundings for LGBTQ+ health centers nationally (51). The number of bills and legislation introduced at the federal and state level continues to steadily increase, and the future health impact of the present political climate on LGBTQ+ patients, particularly younger LGBTQ+ patients, remains to be seen.

Access to health insurance had previously been a barrier for LGBTQ+ patients, but after the Affordable Care Act was passed in 2016, coverage rates between LGBTQ+ patients and their cis-gender peers were observed to be comparable (52). However, disparities exist within the LGBTQ+ community, where LGBTQ+ POC are more like to be uninsured compared to white LGBTQ+ patients (53). LGBTQ+ patients also have higher rates of self-reported financial stressors when accessing healthcare compared non-SOGI minority peers (54).

Despite the growing body of literature demonstrating poorer health outcomes for LGBTQ+ patients, there is wide variation in how medical schools in the United States educate students on these topics (55). Roughly one third of allopathic and osteopathic medical schools in the United States and Canada reported having zero hours of clinical education for students on LGBTQ+ related health issues; the median national average for Canadian and US medical schools was 5 h of training over four years (55). Growing data has begun to emerge that both undergraduate and graduate medical education related to LGBTQ+ is not sufficient to address the unique health needs of LGBTQ+ patients (56). For example, a recent survey of internal medicine residents in 120 programs across the Unites States showed that nearly half of trainees have very limited knowledge of basic knowledge related to LGBTQ+ patients (57). Moreover, medical residents and fellows in the US feel less comfortable discussing LGBTQ+ patient related concerns compared to US medical students (58), which is somewhat concerning. It’s noteworthy that LGBTQ+ medical residents in the US face higher levels of discrimination and bullying by peers and attendings during their medical training compared to their non-SOGI minority peers (59). Some have advocated for a broader commitment by the Accreditation Council for Graduate Medical Education (ACGME) to better incorporate LGBTQ+ health issues pertinent to the residency specialties during graduate medical education training (56).

## 5 LGBTQ+ health in Northern Virginia

The DMV (Washington, District of Columbia, Maryland, and Virginia) area has historically been home to a large and vibrant LGBTQ+ community. However, many health resources dedicated to this population are located within the District of Columbia for several reasons, including geographical concentration of people and centralized distribution of care. As the DMV region continues to grow and expand, it became clear that the LGBTQ+ community in Northern Virginia was lacking in resources specifically dedicated to them within the Virginia region. This need has been a well-known fact highlighted in the findings of several population needs surveys conducted by health departments and local health systems. In June of 2022, Inova Health System opened its first LGBTQ+ clinic named the Inova Pride Clinic (60). The Inova Pride clinic serves LGBTQ+ youths (ages 12 and greater) and adults alike, with over 1,000 new patients in its first calendar year of opening. The purpose of this specialized clinic is to provide comprehensive holistic primary care with an LGBTQ+ focus—to expand the scope of service of primary care to include specific needs for the LGBTQ+ population including gender affirming care, robust mental health services, sexual health, HIV prevention and treatment, and management of chronic diseases.

The clinic has met great success—surpassing initial projections for growth and highlighting the dire need and desire for these services in Virginia and beyond. The Pride Clinic houses medical specialties including internal medicine, pediatrics, obstetrics and gynecology, as well as behavioral health services. Our primary patient population resides in Northern Virginia, Washington, DC, and Maryland. As our clinic continues to grow, we have started to see patients well outside of our geographical location, including the Deep South. Not all patients have the means or resources to travel out of state for medical care, if such medical care is limited in their home state.

At Inova Schar Heart and Vascular, we are working to build a partnership with the Inova Pride Clinic in an effort to enhance cardiovascular care for this community. One of the largest present limitations of cardiovascular research on LGBTQ+ patient's unique health profiles is a lack of SOGI data incorporated into electronic medical records. Going forward, we hope to leverage this robust, comprehensive patient dataset to better understand unique cardiovascular factors impacting LGBTQ+ patients. Long term, we seek to develop the first prospective cardiovascular cohort study of LGBTQ+ patients specifically focused on both clinical and physiologic stressors of health to better understand cardiovascular disease risk factors in this community. Ultimately, we see this as a step towards a broader promotion of community-level LGBTQ+ cardiovascular health equity in the DMV, and especially Northern Virginia.

## 6 Conclusions

Research has only begun to reveal the disparities that LGBTQ+ identifying individuals may face, limiting our understanding of mechanisms underlying these disparities. The purpose of this review was to frame our current understanding of these risks and to consider how to fill existing gaps in knowledge. By having a dedicated LGBTQ+ center of care, we are building a platform which directly allows us to promote community health equity and reduce barriers to care (to the extent that we, as a health system, have control over said barriers). More research, both in the basic science and longitudinal clinical studies, needs to be performed to community to continue using such venues to partner with the community and better learn about their specific needs and most successful strategies to improve their health and improve equity. Our current understanding of the unique health factors impacting LGBTQ+ cardiovascular health will likely change as more data becomes available. The long-term effects of the current political on current and future LGBTQ+ health remains to be seen.
